# Fauna and Predator-Prey Relationships of Ettling, an Actinopterygian Fish-Dominated Konservat-Lagerstätte from the Late Jurassic of Southern Germany

**DOI:** 10.1371/journal.pone.0116140

**Published:** 2015-01-28

**Authors:** Martin Ebert, Martina Kölbl-Ebert, Jennifer A. Lane

**Affiliations:** 1 Jura-Museum Eichstätt, Eichstätt, Germany; 2 Division of Paleontology, American Museum of Natural History, New York, New York, United States of America; University of Oxford, UNITED KINGDOM

## Abstract

The newly recognized Konservat-Lagerstätte of Ettling (Bavaria), field site of the Jura-Museum Eichstätt (JME), is unique among Late Jurassic plattenkalk basins (Solnhofen region) in its abundant, extremely well preserved fossil vertebrates, almost exclusively fishes. We report actinopterygians (ginglymodins, pycnodontiforms, halecomorphs, aspidorynchiforms, “pholidophoriforms,” teleosts); turtles; and non-vertebrates (echinoderms, arthropods, brachiopods, mollusks, jellyfish, sponges, biomats, plants) in a current faunal list. Ettling has yielded several new fish species (Bavarichthys incognitus; Orthogonikleithrus hoelli; Aspidorhynchus sanzenbacheri; Macrosemimimus fegerti). Upper and lower Ettling strata differ in faunal content, with the lower dominated by the small teleost Orthogonikleithrus hoelli (absent from the upper layers, where other prey fishes, Leptolepides sp. and Tharsis sp., occur instead). Pharyngeal and stomach contents of Ettling fishes provide direct evidence that Orthogonikleithrus hoelli was a primary food source during early Ettling times. Scarcity of ammonites and absence of vampyromorph coleoids at Ettling differ markedly from the situation at other nearby localities in the region (e.g., Eichstätt, Painten, Schamhaupten, the Mörnsheim beds), where they are more common. Although the exact biochronological age of Ettling remains uncertain (lack of suitable index fossils), many Ettling fishes occur in other plattenkalk basins of Germany (e.g., Kelheim) and France (Cerin) dated as Late Kimmeridgian to Early Tithonian (eigeltingense horizon), suggesting a comparable geologic age. The Ettling deposits represent an independent basin within the larger Upper Jurassic “Solnhofen Archipelago”, a shallow subtropical sea containing scattered islands, sponge-microbial and coral reefs, sandbars, and deeper basins on a vast carbonate platform along the northern margin of the Tethys Ocean.

## Introduction

The Upper Jurassic Plattenkalk region of Bavaria, Germany is famous for its well preserved fossils [1], [2], including numerous vertebrates (notably *Archaeopteryx*, [3]). The Upper Kimmeridgian to Lower Tithonian limestones of this area formed on the floor of a shallow, subtropical carbonate sea containing scattered islands, sponge-microbial and coral reefs, carbonate sandbars, and deeper basins, on a vast carbonate platform (the “Solnhofen Archipelago”) along the northern margin of the Tethys ocean [1], [2]. Paleogeographically, these plattenkalks or lithographic limestones formed in small (under 10 km in diameter) basins (German: “Wannen” [4], [5]) on the carbonate platform, in the midst of a surrounding shallow epicontinental sea [5], [6], [7], [8]. Although traditionally the basins were considered to have been surrounded by large reefs, later hypotheses have suggested that they were often surrounded not by sponge-algal or coral reefs but by buildups of carbonate sands, which periodically became partially exposed subaerially to form islands ([1], [2], [5]). The closest mainlands were the Rhenish Massif to the north and the Bohemian Massif to the west [1], [2]. Recent studies (see [9], [10], [11]) show that depositional conditions differed between the individual basins, indicating that a generalized approach to depositional setting is insufficient and that each basin should instead be studied on an individual basis to determine its paleoenvironment and depositional setting. This work includes documentation of the Ettling fossil fauna and comparison of the fossil faunas in each basin, which can shed light on paleoenvironmental conditions (e.g., salinity and water depth).

**Figure 1:**
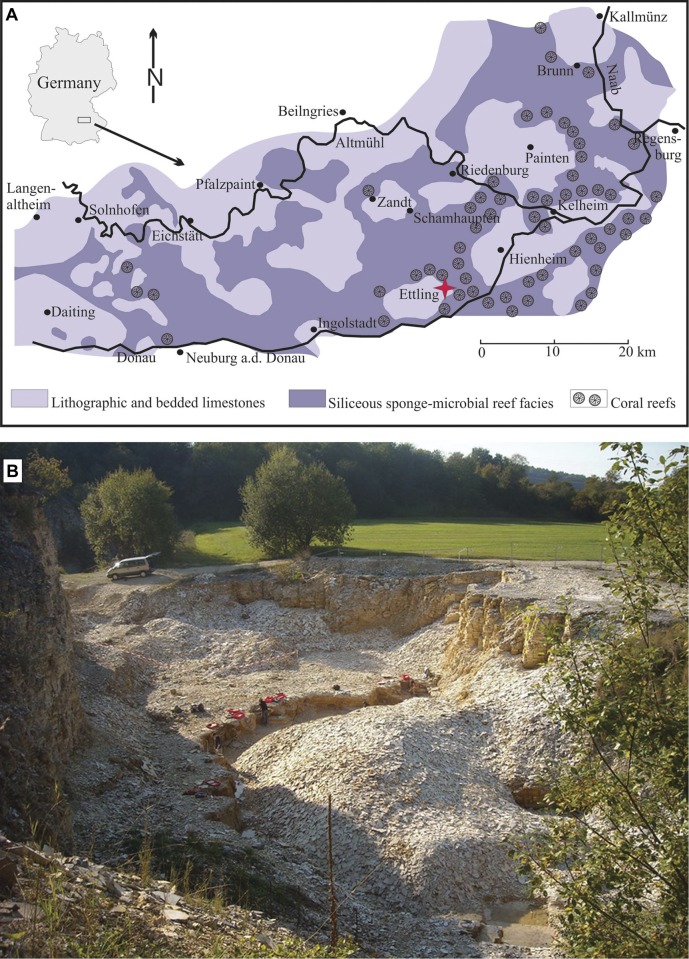
Locality information for the Ettling quarry. A, facies map of the Solnhofen Archipelago of southern Germany, with the locality of Ettling marked by a star, modified from Röper et al., 2000 [11]; B, overview of the Ettling quarry, September 2011, showing the JME field team at work. [Planned for 2-column width.]

The recently discovered Konservat-Lagerstätte of Ettling (Markt Pförring, Bavaria, Germany; Fig. 1), located approximately 50 km E-SE of the village of Solnhofen, is unusual for its excellent preservation and abundance of actinopterygian fishes, which comprise 96% of the total fauna (Tables 1–2). This fish assemblage included several new species (*Orthogonikleithrus hoelli* Arratia, 1997 [12]; *Aspidorhynchus sanzenbacheri* Brito and Ebert, 2009 [13]; *Macrosemimimus fegerti* Schröder et al., 2012 [14]) and even new genera (i.e., *Bavarichthys incognitus* Arratia and Tischlinger, 2010 [15]). Several taxa (e.g., *O*. *hoelli*; *A*. *sanzenbacheri*; *B*. *incognitus*, *M. fegerti*) are currently unique to Ettling and unknown elsewhere. The majority of fossil fishes from Ettling are small forms, with the lower part of the quarry dominated by the small teleost *Orthogonikleithrus hoelli*. Most Ettling species are early teleosts, which first radiated during the Late Jurassic at the time when the Ettling plattenkalks were being deposited. The Ettling locality is therefore of potential importance in understanding the early evolution of modern actinopterygians. The excellent preservation at Ettling also permits answering a variety of important questions regarding paleobiology of fossil actinopterygians, as well as their biogeographical distribution (providing new insight into the breakup of Pangaea) [16–20].

**Table 1:** The Ettling Fauna, Plattenkalks I–III.

**Animalia**									
	**Arthropoda**								
		**Hexapoda**							
			**Insecta**						
				**Odonata**					
					*Stenophlebia*? sp.				2
		**Crustacea**							
			**Decapoda**						
					*Pseudastacus* sp.				1
					*Mecochirus* sp.				1
					*Aeger*? sp. Decapoda indet.				1
									40
			**Ostracoda**						
					Ostracoda indet.				numerous
			**Isopoda**						
					Isopoda indet.				1
	**Brachiopoda**								
		**Terebratulida**							
					Terebratulida indet.				37
		**Rhynchonellida**							
					*Lacunosella* sp.				1
	**Mollusca**								
		**Cephalopoda**							
			**Ammonoidea**						
				**Oppeliidae**					
					Oppeliidae indet.				16
				**Perisphinctoidea**					
					Perisphinctoidea indet.				9
			**Belemnoidea**						
					*Hibolites*?				1
		**Bivalvia**							
					Bivalvia indet.				21
		**Gastropoda**							
					Gastropoda indet.				13
	**Echinodermata**								
		**Echinozoa**							
			**Echinoidea**						
					*Phymosoma* sp.				4
		**Asterozoa**							
			**Ophiuroidea**						
					Ophiuroidea indet.				1
	**Cnidaria**								
		**Hydrozoa?**							
					Hydrozoa? indet.				numerous
	**Vertebrata**								
		**Actinopterygii**							
			**Neopterygii**						
				**Holostei**					
					**Ginglymodi**				
						**Semionotiformes**			
								*Macrosemimimus fegerti*	16
						**Macrosemiiformes**			
								*Notagogus* cf. *denticulatus*	11
					**Halecomorphi**				
						**Amiiformes**			
							**Amiidae**		
								*Amiopsis lepidota*	6
							**Caturidae**		
								Caturidae indet.	1
						**Ionoscopiformes**			
							**Ionoscopidae**		
								*Ionoscopus* sp.	1
							**Ophiopsidae**		
								*Ophiopsis* sp.	4
								*Furo muensteri*	1
				**Pycnodontiformes**					
						*Turbomesodon relegans*			11
						*Proscinetes elegans*			4
						*Proscinetes bernardi*			1
						*Macromesodon* sp.			1
				**Aspidorhynchiformes**					
						*Aspidorynchus sanzenbacheri*			17
						*Belonostomus* cf. *kochi*			20
				**“Pholidophoriformes”**					
						“Pholidophoriformes” n. sp. 1			3
						“Pholidophoriformes” n. sp. 2			1
						*Pleuropholis* sp.			2
				**Teleostei (sensu Arratia, 1999)**					
						*Orthogonikleithrus hoelli*			>3000
						*Orthogonikleithrus* n. sp. 1			59
						*Orthogonikleithrus* n. sp. 2			13
						*Bavarichthys incognitus*			1
						*Pachythrissops* sp.			1
						*Thrissops* cf. *formosus*			42
						Teleostei n. gen. n. sp. 1			89
						Teleostei n. sp. 2			1
		**Sarcopterygi**							
			**Tetrapoda**						
				**Testudines**					
						*Eurysternum*? sp.			2
**Plantae**									
	**Pteridospermales**								
						*Cycadopteris* sp.			1
	**Coniferales**								
						*Brachyphyllum*? sp.			9
**Cyanobacteria?**									
						Biomats			

**Table 2:** The Ettling Fauna, Plattenkalks IV–V.

**Animalia**							
	**Arthropoda**						
		**Hexapoda**					
			**Insecta**				
				**Coleoptera**			
						Coleoptera indet.	1
		**Chelicerata**					
			**Xiphosura**				
						*Mesolimulus* sp.	3
		**Crustacea**					
			**Decapoda**				
						Decapoda indet.	4
	**Brachiopoda**						
		**Terebratulida**					
						Terebratulida indet.	25
	**Mollusca**						
		**Cephalopoda**					
			**Ammonoidea**				
				**Oppeliidae**			
						Oppeliidae indet.	3
				**Perisphinctoidea**			
						Perisphinctoidea indet.	1
		**Bivalvia**					
						Bivalvia indet.	36
		**Gastropoda**					
						Gastropoda indet.	1
	**Echinodermata**						
		**Echinozoa**					
			**Echinoidea**				
						*Phymosoma* sp.	2
		**Asterozoa**					
			**Ophiuroidea**				
						Ophiuroidea indet.	1
	**Cnidaria**						
		**Hydrozoa**					
						Hydrozoa indet.	1
	**Vertebrata**						
		**Actinopterygii**					
			**Neopterygii**				
				**Holostei**			
					**Ginglymodi**		
						*Macrosemius* n. sp.	1
						*Notagogus* cf. *denticulatus*	1
					**Halecomorphi**		
						*Ophiopsis procera*	1
						*Furo muensteri*	1
				**Pycnodontiformes**			
						*Turbomesodon* cf. *relegans*	1
						*Proscinetes bernardi*	1
				**“Pholidophoriformes”**			
						“Pholidophoriformes” sp. 3	1
				**Teleostei (sensu Arratia, 1999)**			
						*Tharsis* n. sp.	2
						*Leptolepides* n. sp.	13
						*Orthogonikleithrus* n. sp. 1	1
						*Thrissops* cf. *formosus*	9
						*Allothrissops mesogaster*	20
**Plantae**							
			*Brachyphyllum*? sp.				6
**Cyanobacteria?**							
			Biomats				

The purpose of the present paper is to provide an overview of the Ettling quarry and a complete list of the Ettling fauna known to date; to present a preliminary investigation of predator-prey relationships and trophic hierarchies during early and late Ettling times based on direct fossil evidence from stomach and pharyngeal contents; and to discuss the potential systematic and paleoecological implications of these new findings.

### Geological setting

**Overview of the Ettling quarry.** The village of Ettling (Markt Pförring) is located on the southernmost rim of the southern Franconian Alb (Fig. 1A) (N 48° 49’ 3’’, E 11° 39’ 34’’). The quarry (Fig. 1B), west of the village, was considered devoid of fossils [21] (except ichnofossils, [22]) until fossil fishes were recently reported by private collectors [25]. A preliminary investigation by JME personnel (2005–2006) revealed numerous diverse actinopterygians, and in 2007 a contract was made with the local community granting the JME permission to conduct scientific excavations at Ettling.

**Figure 2:**
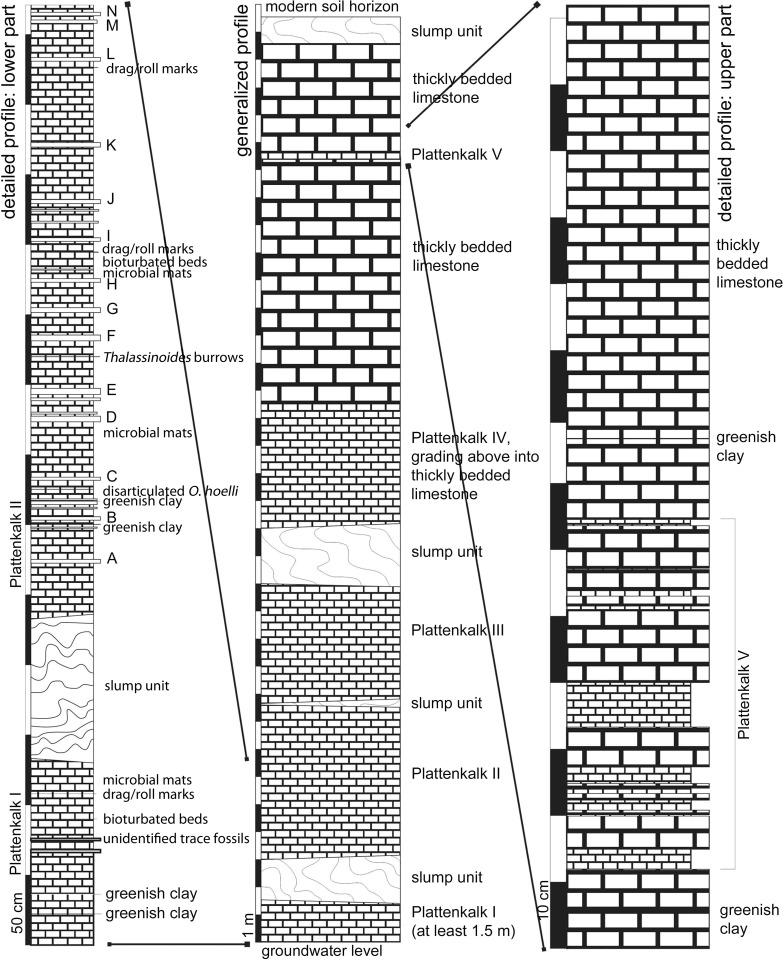
Stratigraphy of the Ettling quarry. Center column, generalized profile of the entire quarry sequence; left column, detailed profile of the excavation site in the lower part of the quarry (containing Plattenkalks I and II); right column, detailed profile of the excavation site in the upper part of the quarry (containing Plattenkalk V). [Planned for 2-column width.]

The Ettling quarry exposure is approximately 33.5 m in vertical height and dissected by numerous vertical tectonic faults (with displacement of up to 30 cm) and joints. The stratigraphic sequence is coarsely subdivided by four slump units (Fig. 2, central column). The lowermost plattenkalk layer (Plattenkalk I: Fig. 2) is approximately 1.5 m thick and located immediately beneath the lowermost slump unit (which is 1 m thick). Immediately above the lowermost slump unit are Plattenkalks II and III (approximately 10 m thick), containing bands of massive limestone beds up to 5 cm in thickness and spaced approximately 20–60 cm apart (e.g., Fig. 2, left column, A–N). These bands can be mapped continuously throughout the quarry and act as useful marker horizons. The second, thin slump unit, which divides Plattenkalks II (below) and III (above), is situated in the middle of this 10 m stack of plattenkalk. The upper boundary of Plattenkalk III is formed by the very conspicuous third slump unit, which is up to 2 m thick (Fig. 2, central column). Following above this upper slump unit are additional plattenkalk layers (Plattenkalk IV) harder than those in the lower part of the quarry. Towards the top of the sequence, the intercalated layers of homogeneous, massive limestone beds increase in thickness and number until the plattenkalk eventually grades into thickly bedded, homogeneous limestone, with individual beds up to 60 cm thick and sometimes separated by millimeter-thin layers of greenish clay. Thin plattenkalk layers (Plattenkalk V) are present within the thickly bedded limestone in the uppermost part of the quarry profile (Fig. 2). Between the massive limestone beds of various thickness, within a space of about half a meter, eight layers of plattenkalk are visible (see detailed profile, Fig. 2, right column), each between a few millimeters and a few centimeters thick. Approximately four meters above the upper layers of Plattenkalk V is a fourth slump unit, before the profile is truncated by the modern soil horizon.

**Figure 3:**
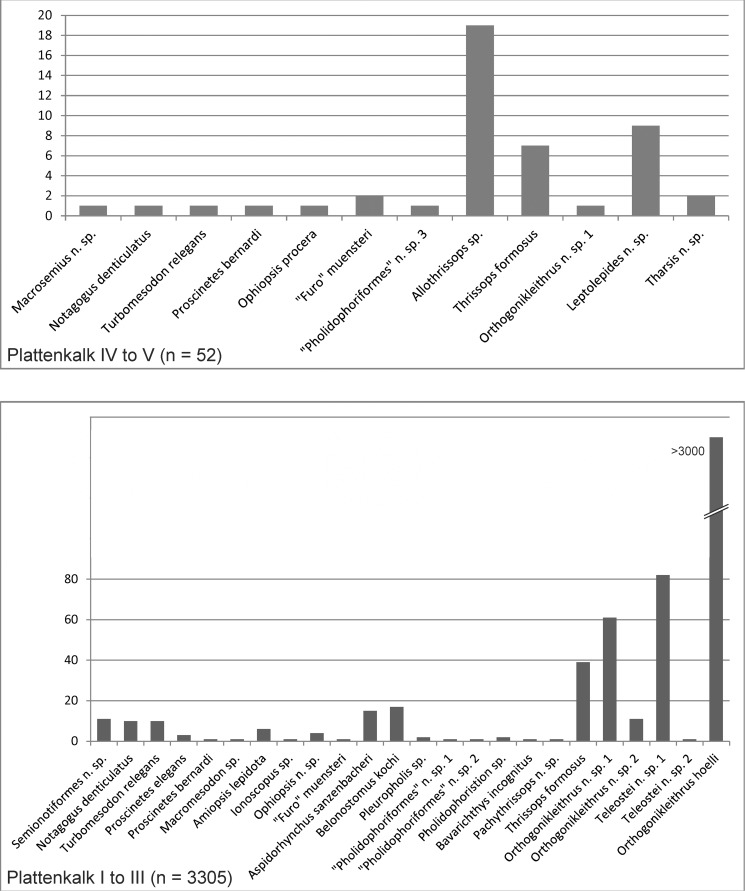
Taxonomic diversity of fishes at Ettling. Data (in the form of total numbers of specimens collected) is given for both the upper (Plattenkalks IV–V) and lower (Plattenkalks I–III) parts of the Ettling quarry (based on data as of March 2012). N = total number of specimens excavated, for each part of the quarry. Over 3000 specimens of *Orthogonikleithrus hoelli* were recovered. [Planned for 2-column width.]

Plattenkalks IV–V (Fig. 2) differ notably in faunal content from Plattenkalks I–III, suggesting a major paleoecological change that took place in what is now a few meters of sediment. The difference is most pronounced in the most common fishes of Plattenkalks I–III vs. Plattenkalks IV–V. Whereas the lower plattenkalks (I to III) are vastly dominated by the small teleost *Orthogonikleithrus hoelli*, this fish is absent from Plattenkalks IV and V, where the genera *Leptolepides*, *Allothrissops* and *Tharsis* (all absent from Plattenkalks I–III) are most common (Tables 1–2; Fig. 3). To reflect this major difference in faunal content, we subdivide the Ettling sequence into a lower part (Plattenkalks I–III) and an upper part (Plattenkalk IV–V). Although Plattenkalk IV (Fig. 2) remains poorly known due to inaccessibility of the steep quarry face, there is thus far no evidence of *O*. *hoelli* in this layer, whereas *Leptolepides* and *Allothrissops* already occur near its lower boundary.

Despite the vast numbers of *Orthogonikleithrus hoelli* within Plattenkalks I–III, these specimens appear randomly distributed, and there are no examples of ‘Fischleinflinze’ (mass accumulations of individuals, of similar sizes, confined to a particular horizon). This finding suggests that they did not result from sudden events resulting in widespread die-offs. This is unlike the situation in the Eichstätt/Solnhofen region, where numerous examples of ‘Fischleinflinze’ are present (e.g., JME-SOS8059). At Ettling, multiple specimens of *Orthogonikleithrus hoelli* are rarely found close to each other on a single bedding plane, and when this does occur the individuals are generally of different sizes, suggesting that they were not members of a single shoal that died simultaneously (unlike the situation in *Leptolepides sprattiformis* from Eichstätt/Solnhofen described by Viohl [23]).

Preliminary sedimentological research in the lower part of the quarry (Plattenkalks I to III) indicates a calcite-dominated primary sediment, suggesting calcitic whitings within a secluded basin ([24], [25]). This distinguishes the lower Ettling plattenkalks from those deposited in the Eichstätt/Solnhofen region [26], [24] (defined as the area containing the Eichstätt and Solnhofen basins [27]). In Plattenkalk V, however, intense pitting of the microspar crystals indicates a higher aragonite content in the initial sediment compared to Plattenkalks I–III (Fellner, pers. comm. 2011; [24]). This slight shift in mineralogy suggests a gradual opening of the basin and greater exposure to influx of external sediments. Unlike the basins of Eichstätt and Solnhofen, there is no evidence at Ettling of differential diagenesis [26], [24].

The diagenetic recrystallization common in plattenkalks from Eichstätt and Solnhofen is not observed in the Ettling plattenkalks [26]; [24], contributing to the higher quality preservation of the Ettling fishes. At Ettling, there is also no evidence of concretions beginning to form around the fossils, a process which leads to the so-called “pedestal and socket preservation” (“Sockel-Erhaltung”; [2]: figs. 6.5 and 6.6) typical at Eichstätt. Most Ettling fishes lie completely flat within the bedding plane.

In addition to the finely laminated plattenkalk layers, there are two layers (each up to 1 cm thick) with a chaotic internal structure, marked in the quarry profile as “bioturbated beds” (Fig. 2). These are clearly distinguishable from the layer with *Thalassinoides* burrows (Fig. 2), which can traverse beds several centimeters in thickness without affecting the surrounding rock. There are also irregularly spaced, greenish clay beds of up to 1 mm in thickness that can be mapped throughout the quarry (the most prominent of these are marked in Fig. 2).

Although most bedding planes are completely flat and nearly smooth, a few contain macroscopic sedimentary structures. Three bedding planes with current indicators (e.g., drag marks and roll marks not caused by ammonites) are present (Fig. 2). Microscopic examination shows that such bedding planes (Fig. 2, left column between H and I) sometimes also contain numerous microscopic echinoderm fragments (ophiuroid sclerites and/or echinoid fragments), small gastropod molds (under 0.5 mm), or microscopic fish remains. A single bedding plane containing completely disarticulated *Orthogonikleithrus hoelli* (a small teleost) has also been observed. Numerous microbial mats (hundreds if not thousands) are distributed throughout the quarry plattenkalks, and are especially prominent on several bedding planes (see Fig. 2).

**Fossils and taphonomic features.** The majority of Ettling fossils are nearly complete fishes preserved in lateral view. Much of the phosphatic skeletal material from Ettling is extremely well preserved, and undisturbed by recrystallization, calcite precipitation, or dendrite formation (typical in fossils from other Solnhofen Plattenkalk localities, e.g., Eichstätt). Large calcite crystals, often observed contacting fish fossils and coprolites from Eichstätt and Solnhofen, are absent in Ettling fossils, and silicification (typical at the nearby locality of Schamhaupten) is also absent. Dendrites are much smaller and rarer than in rocks from the Eichstätt/Solnhofen region.

**Geological age.** The Ettling quarry is part of a larger plattenkalk basin (which has been previously referred to as the “Hartheim Basin”, [21]; see also [28], [29]), whose exact morphology and extent of contact with other plattenkalk basins is unknown [30], [31]. The margins of this larger basin may have been formed at least in part by coral reefs, particularly in the north and east ([7]; Fig. 1). Approximately 1 km north of the quarry the facies grade into reef dolomite; however, presence of a loess cover makes precise mapping of these rocks difficult. A survey of other quarries in the vicinity of Ettling shows that the fish-bearing plattenkalk is restricted to the Ettling quarry. Towards the east these specific beds have been lost due to erosion, and towards the south and west, they are covered by the homogeneous thicker limestone beds that dominate the upper part of the Ettling profile. In other quarries within the Hartheim basin (such as the quarry south of Hagenstetten), these homogeneous thick limestone beds may reach several tens of meters (e.g., 25 m in the infilled quarry of Unterdollig, [21]), interrupted by a number of synsedimentary slump units. The homogenous, thick limestone beds are capped by occasionally bituminous finely bedded marls and marly limestone beds. Such bituminous marls are currently exposed at the quarry of Pettling, where preliminary research shows numerous small teleosts (*Leptolepides* sp.), and more rarely, invertebrates such as brachiopods and ophiuroids (M. Ebert, pers. obs.).

The geological map of the southern Franconian Alb (1:100,000, [32]) assigns the Ettling quarry and its environs to the Solnhofen Formation, a lithostratigraphic unit defined for the Eichstätt/Solnhofen region only [27], comprising thin-bedded plattenkalk as well as thicker limestone beds (‘Bankkalk’). This assignment was based on lithostratigraphic reasoning ([15], [21]; [28], [29], [33], [34]; [35]), a method that leads, however, to major inconsistencies [9]. The current excavation at Ettling has not provided sufficient evidence to confirm its correlation with the Solnhofen Formation. Due to insufficiency of biostratigraphic index fossils (G. Schweigert, pers. comm.), the precise relative age of Ettling remains uncertain. However, many Ettling fish species also occur in other German plattenkalk basins (e.g., Kelheim, especially the Kapfelberg plattenkalk beds) and France (Cerin) dating to the Late Kimmeridgian ([36]; [37]; [38]; Schweigert, pers. comm. 2011; up to the *eigeltingense* horizon, lowermost horizon of the Tithonian [9]), suggesting a potentially similar geologic age (i.e., somewhat older than the plattenkalks of Eichstätt and Solnhofen).

## Results

### Fossil assemblage

**Figure 4:**
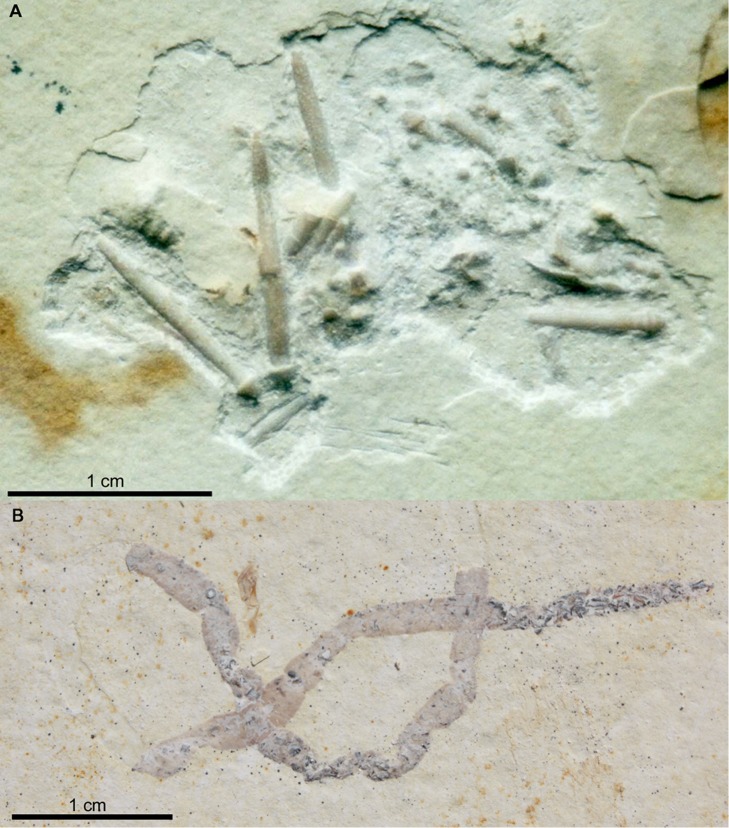
Coprolites from Ettling. A, coprolite or food remains (JME-ETT210) containing echinoid fragments, from Plattenkalk V at Ettling; B, coprolite (JME-ETT3196) containing vertebrae of a small teleost fish (most likely *Orthogonikleithrus hoelli*), from Plattenkalk I at Ettling. [Planned for 1.5-column width.]

**Coprolites and ichnofossils.** Coprolites (Fig. 4) are numerous at Ettling. In Plattenkalks IV–V, only approximately 50% of the coprolites are calcium phosphate, with the other half comprising crushed echinoids and ophiuroids (Fig. 4A). All examined coprolites from Plattenkalks I–III at Ettling (Fig. 4B) contain only fish remains, and over 99% are preserved as calcium phosphate. Their phosphatic composition results in the same dark coloration seen in the vertebrate fossils, making the contents easily observable. A few of these coprolites contain minute teeth of actionopterygian fishes (e.g., pycnodontids: JME-ETT1895; small teleosts: JME-ETT2545). *Thalassinoides* ichnofossils (formed by crustaceans), and other ichnofossils of unknown origin, are known from Plattenkalk II (Fig. 2). Presence of *Thalassinoides* indicates a shallow marine setting ([39]: p. 62).

**Figure 5:**
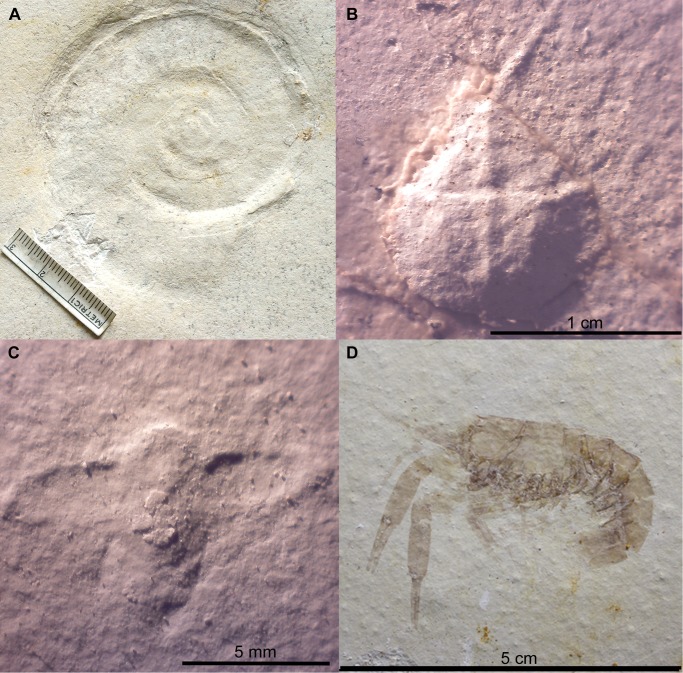
Fossil invertebrates from Ettling. A, an ammonite (Perisphinctidae indet., JME-ETT568a, from Plattenkalk IV), scale bar in millimeters; B, a juvenile horseshoe crab (*Mesolimulus* cf. *walchi*, JME-ETT257a), from Plattenkalk V; C, an unidentified beetle with elytra extended (Coleoptera indet., JME-ETT260, from Plattenkalk V); D, a small crustacean (*Pseudastacus* sp., JME-ETT38a, from Plattenkalk I). [Planned for 2-column width.]

**Non-vertebrate fossils.** Non-vertebrates from Ettling include arthropods (insects, horseshoe crabs, and crustaceans), brachiopods, mollusks, echinoderms, jellyfish, sponges, biomats, and plant fragments (Tables 1–2; Fig. 5). Unlike fishes, most are poorly preserved. Mollusks are rare. Cephalopods comprise only 29 ammonites (impressions in matrix referred to Oppeliidae and Perisphinctoidea, from both the upper and lower Plattenkalk series), and one belemnite with an intact phragmocone (*Hibolites*, from Plattenkalk II). Ammonite aptychi are unknown at Ettling, unlike Eichstätt where they are often found in situ. Bivalves and gastropods are infrequent and poorly preserved, and are known from both Plattenkalks I–III and IV–V. Small gastropods (under 1 cm) occur as molds, and larger specimens (1–2 cm) as impressions in matrix.

Arthropods from Ettling comprise insects (Coleoptera: small beetles, and Odonata: dragonflies resembling the genus *Stenophlebia* from Plattenkalks I–III), xiphosurans (horseshoe crabs), and crustaceans. The latter are represented at Ettling by at least three decapod genera (*Pseudastacus* sp., *Mecochirus* sp., and a specimen resembling *Aeger* from Plattenkalks I–III; and a Decapoda indet. from Plattenkalks IV–V; G. Schweigert, pers. comm.), poorly preserved ostracods (from Plattenkalks I–III), and one indeterminate isopod (also from Plattenkalks I–III). Three specimens of the xiphosuran genus *Mesolimulus* have been found, thus far limited to Plattenkalks IV–V. They are juvenile specimens ranging from one to five cm body length.

Echinoderms are very rare in the lower plattenkalks (I–III) at Ettling, and thus far include only one identified echinoid (*Phymosoma* sp.) and a small Ophiuroidea indet. In Plattenkalks IV–V complete echinoids and ophiuroids are also rare, but here they are often found as fragments within coprolites (see above). The high-magnesium calcite of echinoderms is well preserved. Presence of echinoderms indicates normal marine salinity ([40]: p. 223). Other non-vertebrates from Ettling include brachiopods (several terebratulids from Plattenkalks I–III and IV–IV; one rhynchonellid belonging to *Lacunosella* sp. from Plattenkalk II), sponges (Plattenkalk I), and jellyfish (Plattenkalk V). Microbial biomats are also present (Fig. 2; and see above). Because previous research has shown that such microbial mats can play an essential role in taphonomy and preservation of fossils (as fossils trapped by them are often preserved in pristine condition) [41], the role of biomats at Ettling remains an important topic for future research. Plant fossils thus far comprise two clear leaf imprints of the pteridosperm (seed fern) genus *Cycadopteris* (one from Plattenkalk I and the other from Plattenkalk V), and five poorly preserved imprints of conifer twigs (genus *Brachyphyllum*, from Plattenkalks I–III and IV–V).

**Figure 6:**
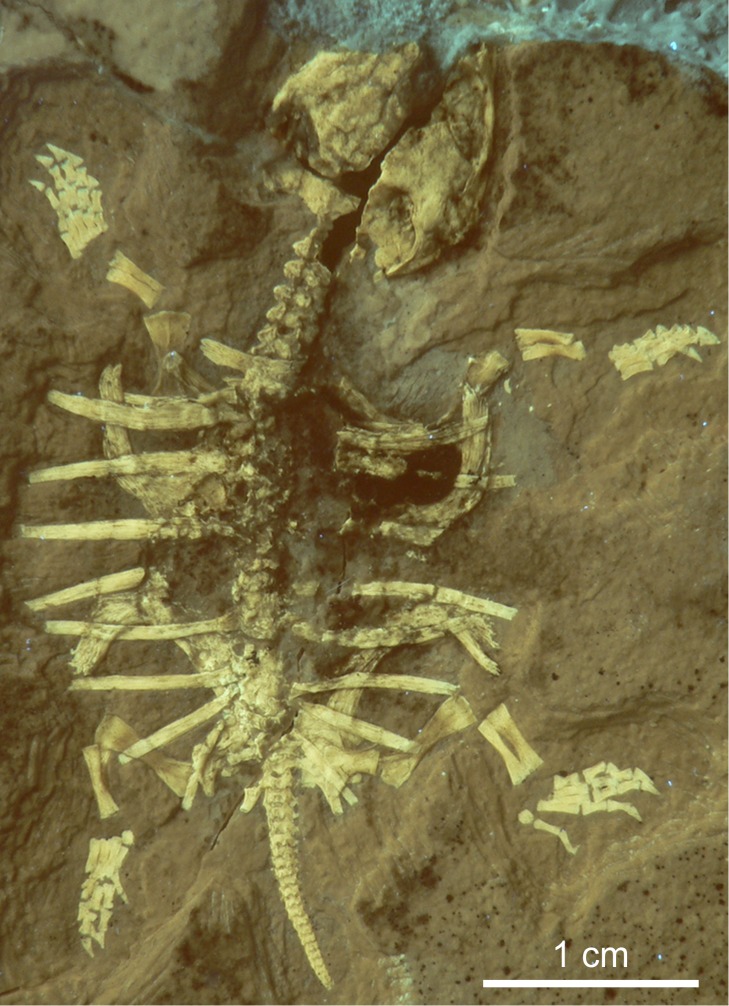
Juvenile turtle from Ettling. Testudinata indet. (possibly *Eurysternum*) (JME-ETT115, from Plattenkalk I), with an unossified carapace suggesting a very early stage of ontogeny. The specimen is shown under UV light, which improves visual contrast between the bone and the matrix (image courtesy of H. Tischlinger). [Planned for 1.5-column width.]

**Vertebrates.** Poorly preserved fragments of an adult turtle were found in Plattenkalk II. A well-preserved juvenile (JME-ETT115, Fig. 6; approximately five centimeters long, with an unossified carapace) was found in Plattenkalk I and tentatively referred to *Eurysternum* sp. [42] (which can attain an adult shell length of over 30 cm).

**Figure 7:**
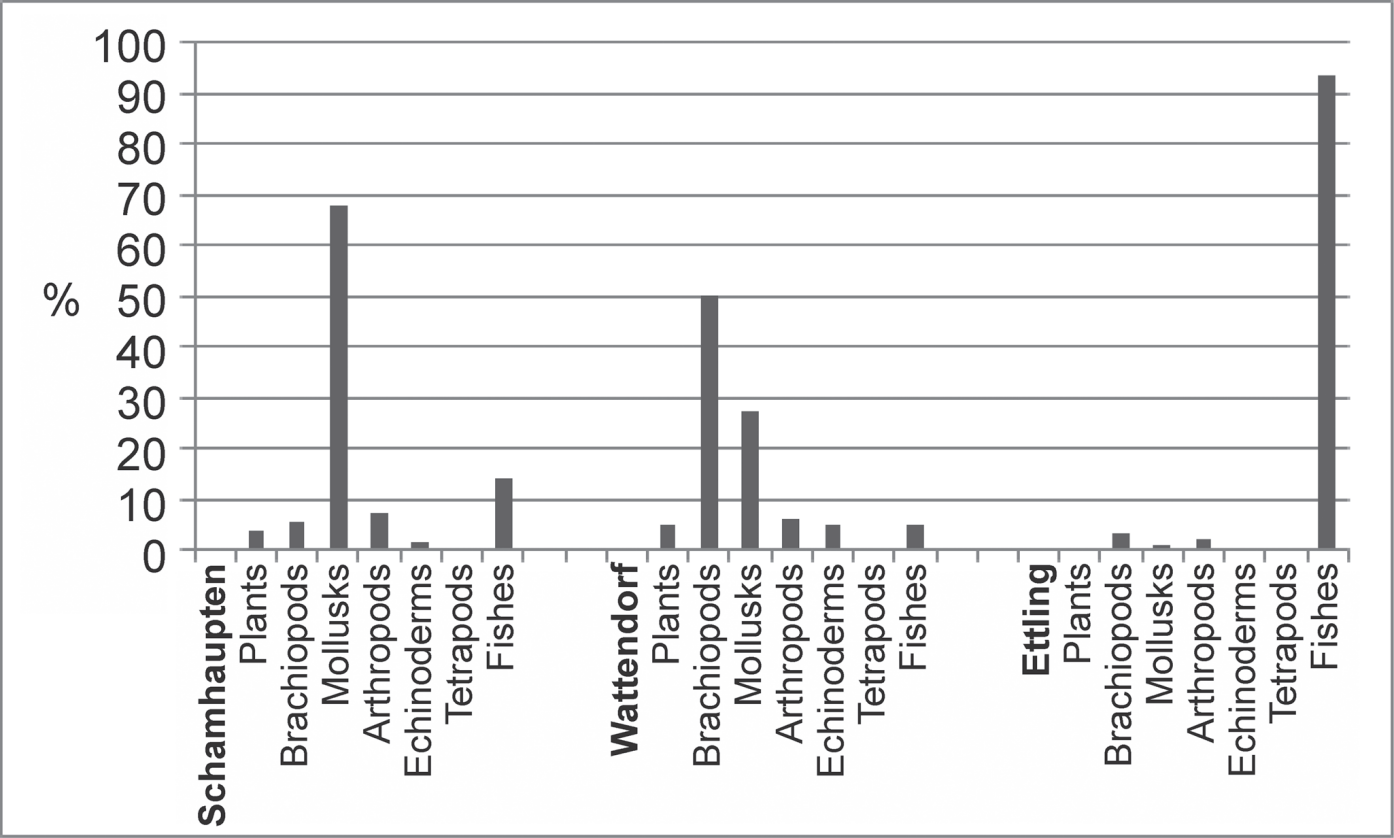
Differing faunal composition of three separate excavation sites in the Solnhofen Archipelago. The locality of Wattendorf is outside of the region shown on the map in Fig. 1, but is located approximately 115 km northwest of Kallmünz (65 km north of Nuremberg). Data for Schamhaupten from [72]; data for Wattendorf from [73]; data for Ettling from the present study. [Planned for 2-column width.]

**Figure 8:**
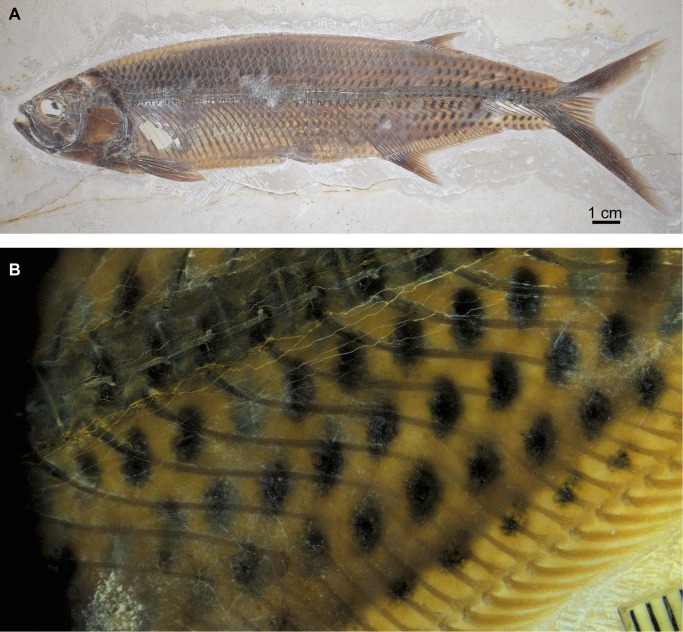
Preservation of color pattern in Ettling actinopterygians. A, *Thrissops* cf. *formosus* from Ettling (JME-ETT74, from Plattenkalk I), overview; B, close-up of the posteroventral region of JME-ETT74 immediately dorsal to the anal fin, showing color pattern preservation (scale bar in millimeters). [Planned for 2-column width.]

At Ettling, actinopterygian fishes comprise 96% of the total fauna recovered (see Fig. 7). They are primarily teleosts, although other taxa are also represented ([12–16], [43–46]). A complete current taxonomic list of fishes from Ettling is provided in Tables 1 and 2, along with the absolute abundance of each species recovered to date. In some of the Ettling fishes, color pattern is preserved (e.g., *Thrissops* cf. *formosus*, see [43]; Fig. 8; also *Allothrissops* and *Tharsis*). The exquisite preservation of Ettling fishes is further illustrated by specimens of *Turbomesodon relegans* showing fine details of the cranial sensory canals (comparable in quality to acid-prepared specimens; see [47], [48]). Similarly, in several specimens of *Proscinetes* traces of skin and muscles are preserved (Ebert and Kriwet, in progress).

### Fossil Actinopterygians from Plattenkalks I–III

To date, the excavation in Plattenkalks I–III ([30]: Fig. 3) has yielded 23 actinopterygian species, of which ten are considered new (Table 1).

**Figure 9:**
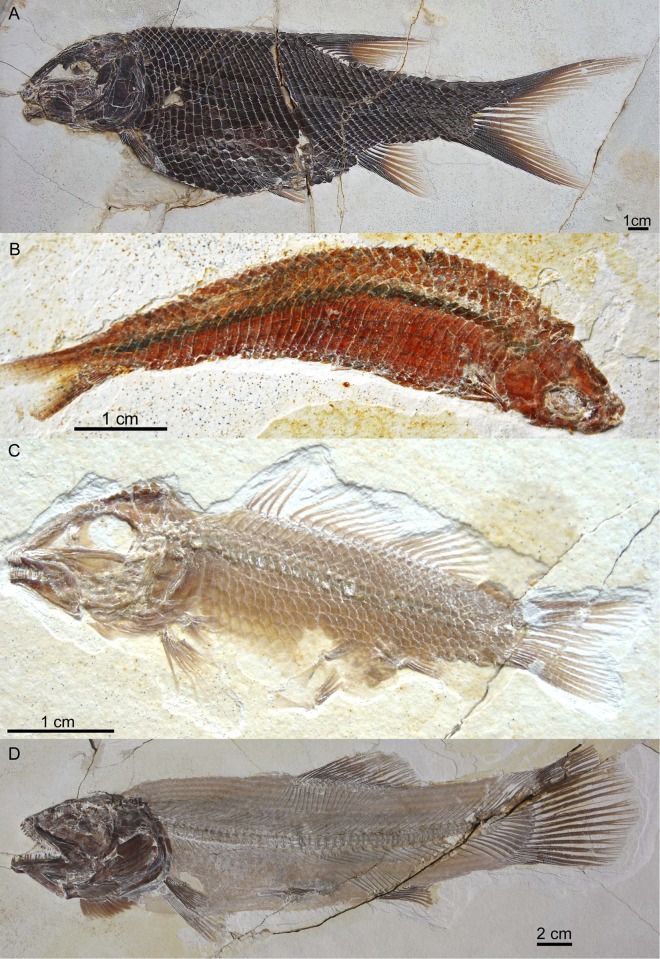
Additional Ettling fishes. A, *Macrosemimimus fegerti* (JME-ETT853, from Plattenkalk II); B, *Pleuropholis* sp. (JME-ETT2980) from Plattenkalk I; C, the macrosemiid *Notagogus* cf. *denticulatus* (JME-ETT116a), from Plattenkalk I; D, *Amiopsis lepidota* (JME-ETT284), from Plattenkalk I. [Planned for 2-column width.]

**Ginglymodins.** Two species of ginglymodins [49] are known from Plattenkalks I–III. One represents a new genus and species of semionotiform (*Macrosemimimus fegerti* Schröder et al., 2012 [14]; Fig. 9), known from 16 specimens. The other is a macrosemiiform (*Notagogus* cf. *denticulatus*: Fig. 9), of which 11 specimens were recovered (representing approximately 4% of non-*Orthogonikleithrus* actinopterygians from Plattenkalks I–III: Table 1). Based on material available in public collections, only 17 specimens of this genus are currently known from other localities in southern Germany (including Brunn, Eichstätt, Kelheim, Painten, and Solnhofen). Of these, *Notagogus denticulatus* Agassiz, 1839 [50] is known only from Kelheim, but is morphologically quite similar to *Notagogus helenae* Thiollière, 1850 [51] from Cerin, France (suggesting possible synonymy with that species).

**Halecomorphs.** Four halecomorph species are currently known from Plattenkalks I–III: one amiid (*Amiopsis lepidota*, Fig. 9), two ophiopsids (*Ophiopsis* sp. [46]; *Furo muensteri*, [45]), and *Ionoscopus* sp. (the latter known only from fragments). The exceptional preservation of the new *Amiopsis* material reveals the presence of fringing fulcra on the dorsal lobe of the caudal fin ([17]: fig. 17), a feature previously unknown in *Amiopsis* and described as absent in amiids (e.g., [52]: p. 847; [53]: p. 583). With sharp, elongate teeth and streamlined fusiform bodies adapted for rapid swimming, halecomorphs such as *Furo muensteri* and *Amiopsis lepidota* were likely among the major predators at Ettling. There is evidence that caturids may also have been present in early Ettling times (represented by a single tooth approximately 1.5 cm in height, JME-ETT231; and a fragmentary specimen in a private collection; both from Plattenkalks I–III), although additional material is needed to confirm this.

**Figure 10:**
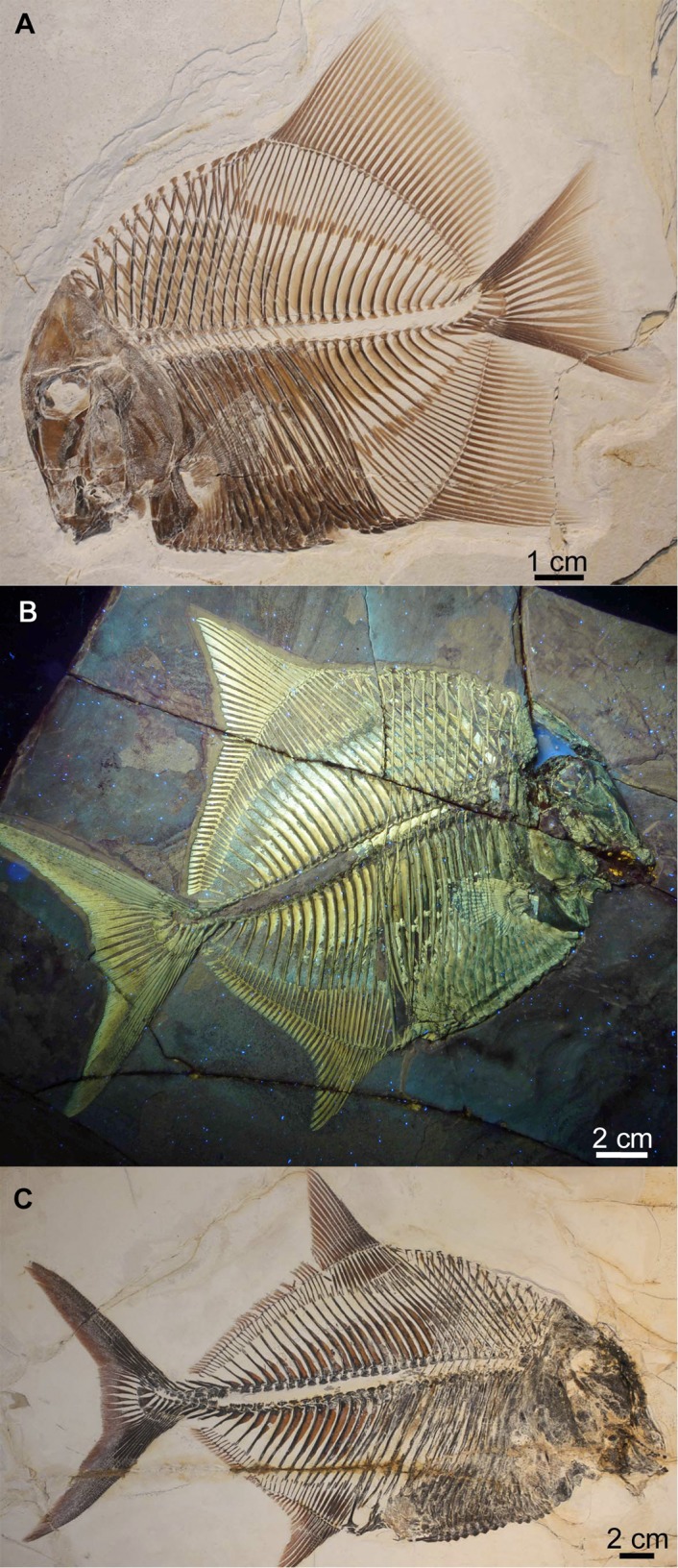
Pycnodontids from Ettling. A, *Turbomesodon relegans* (JME-ETT119), from Plattenkalk I; B, *Proscinetes elegans* (JME-ETT876), from Plattenkalk I, under UV light; C, *Proscinetes bernardi* (JME-ETT250), from Plattenkalk V. [Planned for 1-column width, full page height.]

**Pycnodontiforms.** Four pycnodontiform taxa are currently known from Plattenkalks I–III: *Turbomesodon relegans* (Fig. 10A) *Proscinetes elegans* (Fig. 10B), *Proscinetes bernardi* (Fig. 10C), and *Macromesodon* sp. Of the genus *Proscinetes*, only *P. elegans* was previously known from southern Germany (as well as from Cerin, France [36], [54]). *Proscinetes bernardi* (previously known only from Cerin) has also been recently reported from Kelheim, Bavaria [44]. *Turbomesodon relegans* also occurs in the Eichstätt and Solnhofen basins.

**Figure 11:**
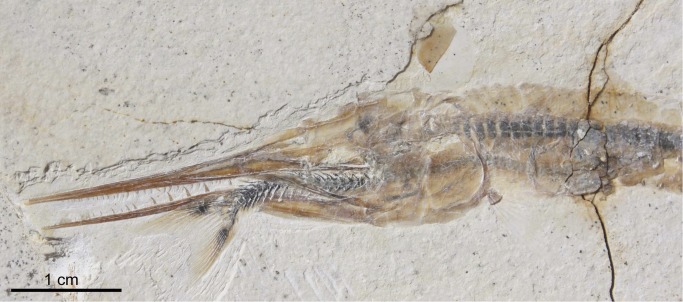
*Belonostomus* cf. *kochi* (JME-ETT123, from Plattenkalk I), with a specimen of *Orthogonikleithrus hoelli* as pharyngeal contents. [Planned for 2-column width.]

**Aspidorhynchiforms.** Plattenkalks I–III contains two aspidorhynchiform species (*Aspidorhynchus sanzenbacheri* and *Belonostomus* cf. *kochi*). *Belonostomus* cf. *kochi* (Fig. 11) is the most common non-teleost fish in Plattenkalks I–III. *Belonostomus kochi* Münster, 1836 [55] was previously known only from Kelheim. This species represents one of a number of similar examples (e.g., *Notagogus denticulatus*, *Proscinetes bernardi*, *Furo muensteri*) found at both Ettling and Kelheim (for a preliminary comparison between the fish fauna of Kelheim, Cerin, Ettling, and Eichstätt, see table 2 in [44]). *Aspidorhynchus sanzenbacheri* Brito and Ebert, 2009 [13] is distinguishable from *A. acutirostris* (found in the Eichstätt and Solnhofen basins) by its noticeably shorter rostrum.

**“Pholidophoriforms”.** The order Pholidophoriformes Berg, 1940 [62] is non-monophyletic [56–61]. Arratia [60] found that within the group, only the European Triassic family Pholidophoridae is monophyletic, forming the sister group to *Eurycormu*s and all other teleosts. Many of the Jurassic forms previously assigned to “Pholidophoriformes” remain unresolved. Two new species of “Pholidophoriformes” are present in Plattenkalks I–III, here referred to as “Pholidophoriformes” n. sp. 1 and “Pholidophoriformes” n. sp. 2 (both currently under study by G. Arratia). A single, complete specimen of *Pleuropholis* sp. (Fig. 9B) and an additional fragmentary specimen from Plattenkalks I–III were also found (Table 1). Pleuropholidae are currently known mainly from the Late Triassic to Early Cretaceous of Europe, Africa, and Brazil [63]. In the Late Jurassic, *Pleuropholis* is represented by three specimens from Eichstätt/Solnhofen and one specimen from the overlying Mörnsheim beds, as well as more numerous specimens from Kelheim (approximately eight specimens in public collections), and Cerin, France (with at least 23 specimens). Pleuropholids have been identified by previous authors as basal teleosts (within the “Pholidophoriformes”, [56], [64–66]); however, due to the non-monophyly of this group [56–61], the position of Pleuropholidae is also in need of revision.

**Teleostei.** Early teleosts first appeared in the Late Triassic [12], [56], [57], [60]. They radiated in the Jurassic to produce numerous new taxa, and now comprise approximately 96% of extant fish species [67]. The Ettling fauna adds to knowledge of this important period in teleost evolution by contributing several new taxa.

**Figure 12:**
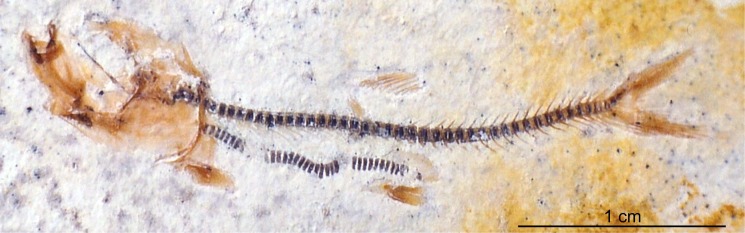
*Orthogonikleithrus hoelli* (JME-ETT129, from Plattenkalk I), containing a smaller conspecific individual as stomach contents. [Planned for 2-column width.]

**Figure 13:**
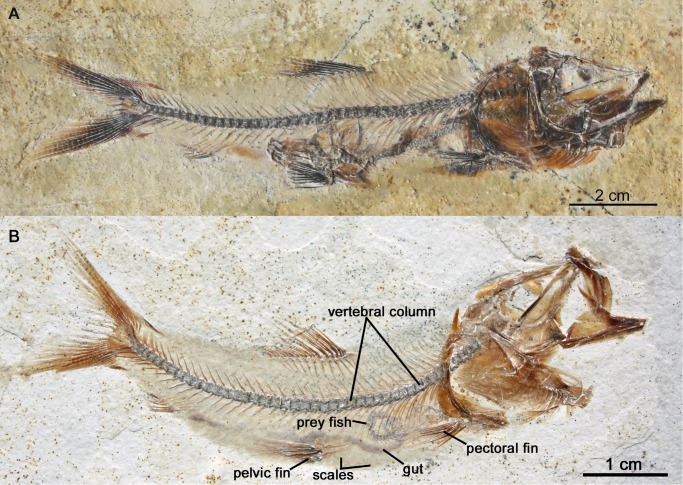
Predation on *Orthogonikleithrus hoelli* by other *Orthogonikleithrus* species at Ettling. A, *Orthogonikleithrus* n. sp. 1 (JME-ETT903), a new species of this genus from Ettling (Plattenkalk II) (Arratia, in progress), with *Orthogonikleithrus hoelli* as stomach contents; B, *Orthogonikleithrus* n. sp.1 (JME-ETT9) from Plattenkalk I), with *Orthogonikleithrus hoelli* as stomach contents. [Planned for 2-column width.]

**Figure 14:**
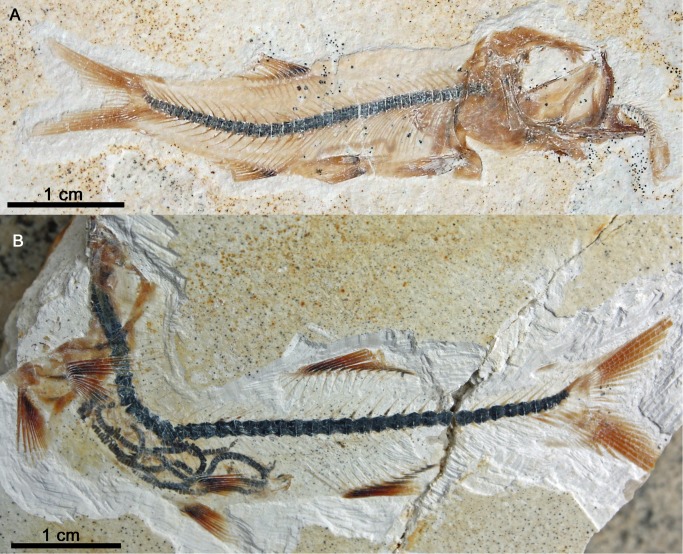
Predation on *Orthogonikleithrus hoelli* at Ettling. A, Teleostei n. sp. 1 (JME-ETT976, from Plattenkalk II), a new teleost from Ettling (Arratia, in progress), containing a specimen of *Orthogonikleithrus hoelli* as pharyngeal contents; B, Teleostei n. sp. 1 (JME-ETT194) from Plattenkalk II, with numerous specimens of *Orthogonikleithrus hoelli* as stomach contents. [Planned for 2-column width.]

Teleostei (sensu Arratia, 2013 [60]) from Plattenkalks I–III comprise *Orthogonokleithrus hoelli* Arratia, 1997 (Euteleostei, Orthogonikleithridae; Fig. 12); at least two new species of *Orthogonikleithrus* (Fig. 13; Arratia, in progress; Konwert, in progress); *Thrissops* cf. *formosus* (Ichthyodectiformes [60]; Fig. 8); *Bavarichthys incognitus* (Crossognathiformes [60]); one new species of *Pachythrissops* (Ichthyodectiformes [60]); and one additional new genus and two species of Teleostei (Arratia, in progress; Fig. 14). *Orthogonikleithrus hoelli* is represented by numerous adult and juvenile specimens [20]. Specimens of *Thrissops* cf. *formosus* show variations in morphology and color pattern, and include an ontogenetic series ranging in length from 4.3 cm (in a largely unossified juvenile; Fig. 12) to a maximum of 55 cm. Arratia and Tischlinger [15] described the new species *Bavarichthys incognitus* from Ettling, representing the earliest crossognathiform fish known from Europe. Crossognathiforms (an extinct order of basal teleosts) are otherwise known in Europe only from the Cretaceous. Older Jurassic crossognathiforms are known from Chile and Cuba. A faunal exchange between areas so widely separated during the Jurassic (the Caribbean/South America and the northern margin of the Tethys) would have been possible via the young Mid-Atlantic [15], [19].

### Fossil Actinopterygians from Plattenkalks IV–V

**Ginglymodins.** Two macrosemiids (one new species of *Macrosemius*, and *Notagogus* cf. *denticulatus*) occur in Plattenkalk V. Each taxon is known only from a single specimen (Table 2).

**Halecomorphs.** Two halecomorphs are present: a single specimen of *Ophiopsis procera* (a different species form from that found in the lower part of the quarry; [46]) from Plattenkalk V, and a single specimen of *Furo muensteri* from Plattenkalk IV ([30], [45]).

**Pycnodontiforms.** Two pycnodontiforms, *Proscinetes bernardi* (Fig. 10C) and *Turbomesodon* cf. *relegans*, are known from Plattenkalk V (each from a single specimen; Table 2). The specimen of *Proscinetes* is extremely well preserved, and represents the largest individual of this genus yet known from the Late Jurassic of southern Germany. The specimen of *Turbomesodon* (JME-ETT145), albeit fragmentary, is the largest representative of its genus yet known (at approximately 25 cm in length).

**“Pholidophoriforms”.** A third new “pholidophoriform” species (which differs from the two species occurring in Plattenkalks I–III) appears in Plattenkalk V (currently under study by G. Arratia).

**Teleosts.** Several Teleostei (sensu Arratia, 2013) [56–58], [60] occur in the upper part of the Ettling quarry (Plattenkalks IV–V), including *Thrissops* cf. *formosus*, which also occurs in the lower part of the quarry. The upper quarry section yields genera typical for previously known localities of the Eichstätt/Solnhofen region, including *Leptolepides* sp., *Tharsis* sp., and *Allothrissops mesogaster* [50] (Fig. 3, upper chart; Table 2). These three genera are absent from Plattenkalks I–III. Probable new species of *Leptolepides* and *Tharsis* from Ettling are currently under study by G. Arratia.

### Fossil evidence of predation in Ettling fishes

A total of 59 actinopterygian fish specimens from Plattenkalks I–III from the JME excavations at Ettling contain fossil prey items. Of these, 20 specimens contain prey lodged in the pharynx, and 39 specimens contain prey in the stomach (Tables 3–4). In all specimens the prey items consisted entirely of fishes, with *Orthogonikleithrus hoelli* confirmed as prey in 53 cases and considered probable prey (based on vertebral morphology) in five of the remaining six cases. Only one specimen, JME-ETT11 (Table 4), contains a prey fish other than *O*. *hoelli*.

**Table 3:** Direct fossil evidence of predation in Ettling actinopterygians: predators with prey lodged in the pharynx (SL = standard length).

**Species name**	**Specimen number**	**SL (predator)**	**Pharyngeal contents**	**SL (prey fish)**
*Belonostomus* cf. *kochi*	JME-ETT123a	12.5 cm (juvenile)	*Orthogonikleithrus hoelli*	~4.5 cm
*Orthogonikleithrus hoelli*	JME-ETT106	3.5 cm	*Orthogonikleithrus hoelli*	~1 cm
	JME-ETT174	4 cm	*Orthogonikleithrus hoelli*	~2 cm
	JME-ETT175	3 cm	*Orthogonikleithrus hoelli*	indet.
	JME-ETT571	3.5 cm	*Orthogonikleithrus hoelli*	~2 cm
	JME-ETT2642	2 cm	*Orthogonikleithrus hoelli*	~1 cm
Teleostei n. sp. 1	JME-ETT4	4.5 cm	*Orthogonikleithrus hoelli*	~2.5 cm
	JME-ETT24	5.5 cm	*Orthogonikleithrus hoelli*	~3.5 cm
	JME-ETT27	4.5 cm	*Orthogonikleithrus hoelli*	~2 cm
	JME-ETT54	5.5 cm	*Orthogonikleithrus hoelli*	~3 cm
	JME-ETT167	skull length 2 cm	*Orthogonikleithrus hoelli*	~2.5 cm
	JME-ETT176	4.5 cm	*Orthogonikleithrus hoelli*	~2.5 cm
	JME-ETT277	6.5 cm	*Orthogonikleithrus hoelli*	~3 cm
	JME-ETT901	skull length 2 cm	*Orthogonikleithrus hoelli*	~2.5 cm
	JME-ETT976	5 cm	*Orthogonikleithrus hoelli*	~2.5 cm
	JME-ETT982	7.0 cm	*Orthogonikleithrus hoelli*	~3 cm
	JME-ETT1362	6.0 cm	*Orthogonikleithrus hoelli*	~2.5 cm
	JME-ETT1535	5.0 cm	*Orthogonikleithrus hoelli*	~2.5 cm
	JME-ETT1946	3.0 cm	*Orthogonikleithrus hoelli*	~1.5 cm
*Orthogonikleithrus* n. sp. 1	JME-ETT1348	6.5 cm	*Orthogonikleithrus hoelli*	~3 cm

**Table 4:** Direct fossil evidence of predation in Ettling actinopterygians: predators with prey in the stomach (SL = standard length).

**Species name**	**Specimen number**	**SL (predator)**	**Stomach contents**	**SL (prey fish)**
*Belonostomus* cf. *kochi*	JME-ETT893b	indet.	*Orthogonikleithrus hoelli*	~2 cm
	JME-ETT1900	24 cm	*Orthogonikleithrus hoelli*	~2 cm
*Thrissops* cf. *formosus*	JME-ETT166	12.5 cm	*Orthogonikleithrus hoelli*	~2 cm
	JME-ETT93	indet.	*Orthogonikleithrus hoelli*	~2 cm
	JME-ETT2079	33 cm	*Orthogonikleithrus hoelli*	~3 cm
*Orthogonikleithrus hoelli*	JME-ETT104	3.5 cm	*Orthogonikleithrus hoelli*	~2 cm
	JME-ETT111	3.0 cm	*Orthogonikleithrus hoelli*	~1.5
	JME-ETT129	3.0 cm	*Orthogonikleithrus hoelli*	~2 cm
	JME-ETT143	4.0 cm	*Orthogonikleithrus hoelli*	~2 cm
	JME-ETT144	4.5 cm	*Orthogonikleithrus hoelli*	~2 cm
	JME-ETT159	3.0 cm	*Orthogonikleithrus hoelli*	~2 cm
	JME-ETT180	3.5 cm	*Orthogonikleithrus hoelli*	~2 cm
	JME-ETT181	3.5 cm	fish vertebrae (*O. hoelli*?)	indet.
	JME-ETT267	4.5 cm	*Orthogonikleithrus hoelli*	~2 cm
	JME-ETT1459a,b	4.0 cm	*Orthogonikleithrus hoelli*	~2 cm
	JME-ETT1536	4.5 cm	*Orthogonikleithrus hoelli*	~2 cm
	JME-ETT1945	4.5 cm	*Orthogonikleithrus hoelli*	~2 cm
	JME-ETT1954,	4.5 cm	*Orthogonikleithrus hoelli*	~2 cm
	JME-ETT2540	4.5 cm	*Orthogonikleithrus hoelli*	~1.5
	JME-ETT2541	3.5 cm	*Orthogonikleithrus hoelli*	~1.5 cm
	JME-ETT2636	3.5 cm	*Orthogonikleithrus hoelli*	~2 cm
*Orthogonikleithrus* n. sp. 1	JME-ETT9	7.0 cm	fish vertebrae (*O. hoelli*?)	indet.
	JME-ETT30	6.0 cm	fish vertebrae (*O. hoelli*?)	indet.
	JME-ETT31	8 cm	*Orthogonikleithrus hoelli*	~3 cm
	JME-ETT121	6.5 cm	*Orthogonikleithrus hoelli*	indet.
	JME-ETT195	5.0 cm	*Orthogonikleithrus hoelli*	~2 cm
	JME-ETT263	9.0 cm	*Orthogonikleithrus hoelli*	~3.5 cm
	JME-ETT563	5.5 cm	*Orthogonikleithrus hoelli*	indet.
	JME-ETT572	~7 cm	fish vertebrae (*O. hoelli*?)	indet.
	JME-ETT903	6.5 cm	*Orthogonikleithrus hoelli*	~4 cm
	JME-ETT1364	5.5 cm	*Orthogonikleithrus hoelli*	~3.5 cm
	JME-ETT1806	7.0 cm	*Orthogonikleithrus hoelli*	~4 cm
Teleostei n. sp. 1	JME-ETT118	6.5 cm	*Orthogonikleithrus hoelli* (several specimens)	various
	JME-ETT194	7.0 cm	*Orthogonikleithrus hoelli* (several specimens)	various
	JME-ETT132	indet.	*Orthogonikleithrus hoelli*	indet.
	JME-ETT148	skull 2.5 cm	*Orthogonikleithrus hoelli*	~4 cm
	JME-ETT173	6.5 cm	*Orthogonikleithrus hoelli*	~4 cm
	JME-ETT279	4.5 cm	fish vertebrae (*O. hoelli*?)	indet.
	JME-ETT11	6.5 cm	Unidentified small teleost	indet.
*Bavarichthys incognitus*	JME-SOS4934	14 cm	*Orthogonikleithrus hoelli*	~3 cm

Twenty out of a total 3,000 specimens of *Orthogonikleithrus hoelli* contain smaller conspecific individuals as stomach or pharyngeal contents, indicating occasional cannibalism (Fig. 13). Even juveniles (e.g., JME-ETT2642, a specimen of 2.2 cm total length) are found containing smaller fish as prey. In contrast to *Orthogonikleithrus*, specimens of *Leptolepides sprattiformis*, the most common small teleost in the Eichstätt/Solnhofen area, have not been found containing prey items. Fossil evidence from Ettling shows that predators of *O. hoelli* included an additional new species of *Orthogonikleithrus* (G. Arratia, pers. comm.) that is larger than *O. hoelli* (reaching up to 13 cm in length; Fig. 13A). Another small teleost (Teleostei n. sp. 1, Arratia, in progress), which has large teeth, often (in about 15% of all known specimens) contains partially digested or half-swallowed specimens of *Orthogonikleithrus hoelli* (Fig. 14B). In the majority of specimens with pharyngeal contents prey is orientated headfirst (the preferred orientation for gape-limited fish predators; [67]: p. 328; [68]). Specimens with prey lodged in the pharynx are common in the fossil record, and represent occasional errors in judgment by the predators (i.e., selection of too-large prey), resulting in death by choking [23]; [67–70].

Approximately 50% of adult specimens of *Orthogonikleithrus* n. sp. 1 from Ettling, and 67% of adult specimens of Teleostei n. sp. 1, contain prey in the pharynx or stomach. Stomach contents of a single individual sometimes contain more than one *Orthogonikleithrus hoelli* of different sizes and ages (see Fig. 14B; Table 4). The holotype and only specimen of *Bavarichthys incognitus* also contains *O. hoelli* as stomach contents. Much larger predatory teleosts at Ettling, such as *Thrissops* cf. *formosus* [43], which at 55 cm in length is among the largest predatory fishes from this locality, have also been found with *Orthogonikleithrus hoelli* as stomach contents. This predatory species has very small teeth, consistent with a diet of small prey fishes. A small juvenile specimen of *Thrissops* cf. *formosus* with stomach contents (JME-ETT 166; total length 13 cm) also contains *O. hoelli*. Although stomach contents are rarely visible in fishes with ganoid scales (due to their thickness and opacity), a juvenile *Belonostomus* cf. *kochi* from Ettling is preserved with an *Orthogonikleithrus hoelli* lodged in the pharynx (Fig. 11; [16]: Fig. 8) and two other specimens were found with *O. hoelli* in the stomach (see Table 4). These are among the first documented examples of piscivory in *Belonostomus* from Bavaria ([23]: tab. 1).

## Discussion

### Paleoecological comparison of Ettling with other plattenkalk basins

Ettling differs notably in its faunal composition from other well-known plattenkalk localities of the region (e.g., Eichstätt, Wattendorf, Schamhaupten). At Ettling the fauna is predominantly (96%) actinopterygian fishes (Fig. 7), and fossil stomach contents as well as coprolites from Plattenkalks I–III comprise only fish remains (Tables 3–4; Fig. 4B; [16]). In contrast, at Eichstätt invertebrates (e.g., the nektonic to planktonic crinoid *Saccocoma* and small ammonites) are the predominant fossils, so common that some beds are virtually covered with specimens. *Saccocoma* ([2]: p. 74) and aptychi are also common in coprolites from Eichstätt. The Eichstätt basin is additionally characterized by vampyromorph coleoids (e.g., *Plesioteuthis*, *Trachyteuthis*, *Leptoteuthis*, *Muensterella*, *Palaeologio*) [71]) and a diverse arthropod fauna (as well as fish taxa), whereas at Ettling arthropods and ammonites are much rarer, and *Saccocoma* and vampyromorph coleoids are absent.

The Ettling fauna also differs from that of Schamhaupten and Wattendorf, which (unlike Eichstätt) have been the sites of recent scientific studies ([72], [73]; Fig. 7). Unlike Ettling, the fauna at Schamhaupten is 69.6% mollusks (e.g., ammonites), with fishes as a minority (representing 14.2% of known specimens) [72].Vampyromorph coleoids are also present at Schamhaupten (where some specimens contain fish remains as stomach contents [72]). At Wattendorf, invertebrates also outnumber fishes, with brachiopods representing 50% of total specimens (whereas fishes represent 5% of total specimens) [73]. Although a comprehensive statistical comparison of the Ettling fauna with that of other German plattenkalk basins is beyond the scope of this paper, compilation of a database for future statistical analysis has already begun (Ebert and Kölbl-Ebert, in progress).

Ettling differs notably from other localities of the region in its rarity of cephalopod fossils. In addition to the rarity of ammonite shells, the coprolite *Lumbricaria* (attributed to ammonites: [1]: p. 360; [74]: p. 134) is absent at Ettling. In contrast, *Lumbricaria* is common in the Eichstätt and Solnhofen basins (often containing only *Saccocoma*). Among coleoids, belemnites are extremely rare at Ettling, and there is no evidence of vampyromorph coleoids. One specimen of the vampyromorph *Trachyteuthis hastiformis* (JME-SOS8329) from Eichstätt contains multiple *Leptolepides sprattiformis* lacking the heads and caudal fins, interpreted as stomach contents [75]. Evidence of similar feeding habits in other coleoids ([76]: p. 48; including recent octopi: D. Fuchs, pers. comm., 2012), suggests that abundant isolated heads and caudal fins of small fishes (*Leptolepides sprattiformis* and juvenile *Tharsis dubius*) at Eichstätt and Solnhofen (cf. [77]: p. 359) may represent coleoid feeding behavior (cf. [77]: p. 359; [76]: p. 48). In contrast, such isolated fish skulls are not found at Ettling.

In addition to its overall faunal composition, the fish fauna at Ettling also differs from that of other plattenkalk localities of the region. For example, in the Eichstätt basin the teleosts *Leptolepides* Nybelin, 1974 [78], *Tharsis* Giebel, 1848 [79], and *Allothrissops* Nybelin, 1964 [80] are the most common fishes. At Ettling, these genera occur in the upper part of the quarry (*Leptolepides* and *Allothrissops* are relatively common in Plattenkalk V, and also occur in Plattenkalk IV), but not in Plattenkalks I–III. Additionally, the species of *Tharsis* and *Leptolepides* from Ettling are distinct from those found at Eichstätt (Arratia, in progress). The teleost species *Orthogonikleithrus hoelli* is thus far unique to Ettling, and only found in Plattenkalks I–III. This species (so abundant in the lower part of the quarry) is not found above the slump unit underlying Plattenkalk IV.

**Predator-prey relationships at Ettling, and the role of *Orthogonikleithrus hoelli.*** Konservat-Lagerstätten such as Ettling, with their excellent preservation and abundance of fossils, provide a rare glimpse into predator-prey relationships and trophic structure of ancient aquatic communities. Although a complete paleoecological investigation is beyond the scope of the present paper, Maisey [81] showed that much information regarding trophic structure in aquatic paleocommunities can be gleaned from investigating the stomach and pharyngeal contents of fossil fishes. He also outlined the reasons why specimens containing prey items in the pharynx and/or stomach are generally rare in the fossil record [81].

Maisey [81] used the criteria of Boucot [82] to classify the different types of evidence that can be applied to paleoecological studies of fossil fishes. Of these, ‘Category 1’ evidence (the most reliable) comprises ‘frozen behavior’ (e.g., one fish eating another). ‘Category 2’ evidence (slightly less reliable) comprises ‘Category 2A’ (close association but not in position, e.g., presence of both a predator and its presumed prey in the same strata) and ‘Category 2B’ (functional interpretation of morphology, e.g. large robust teeth of a presumed predator). Categories 3, 4, and 5, considered by Boucot [82] to be progressively more speculative, include trace fossils of unknown origin (e.g., trackways). Maisey [81] placed ‘isolated bone balls’ of presumably digested or regurgitated prey (not directly associated with remains of a specific predator) in this category.

Because scientific fieldwork at Ettling was begun only recently (in 2007), it will be some time before a complete picture of its paleoecology can be reconstructed (requiring the discovery of additional fossil specimens and detailed investigation of macroinvertebrates, palynology, data from microfossils, etc.). However, there is already an abundance of reliable ‘Category 1’ [81], [82] evidence of predator-prey relationships (numerous specimens with stomach and pharyngeal contents), from which we can begin to reconstruct fish trophic structure in the Ettling paleocommunity (see Tables 3–5). ‘Category 2’ evidence is also present (for example, Category 2A: presence of *Thrissops* and *Leptolepides* together in Plattenkalk V, two genera previously known from direct evidence to have been predator and prey, respectively, at other plattenkalk localities: [81]; Category 2B: presence of large, robust, sharp teeth in *Furo muensteri* suggesting that it was a major predator: [45]).

**Table 5:** Probable trophic levels of actinopterygian fishes at Ettling. Other*: probable suction-feeding micro-carnivores/visual zooplanktivores and opportunistic piscivores.

High-level predators	Caturidae indet.
	*Ionoscopus* sp.
	*Amiopsis lepidota*
	*Furo muensteri*
	*Aspidorynchus sanzenbacheri*
Mid-level predators	*Ophiopsis procera*
	*Ophiopsis* sp.
	*Pachythrissops* sp.
	*Thrissops* cf. *formosus*
	*Bavarichthys incognitus*
	*Belonostomus* cf. *kochi*
	“Pholidophoriformes” sp. 1
	“Pholidophoriformes” sp. 2
Low-level predators	*Orthogonikleithrus* n. sp. 1
	*Allothrissops mesogaster*
	Teleostei n. sp. 1
	Teleostei n. sp. 2
	“Pholidophoriformes” sp. 3
	*Macrosemius* n. sp.?
	*Notagogus* cf. *denticulatus*
	*Pleuropholis* sp.
Durophages	*Turbomesodon relegans*
	*Proscinetes bernardi*
	*Proscinetes elegans*
	*Macromesodon* sp.
	*Macrosemimimus fegerti*
Other*	*Orthogonikleithrus hoelli*?
	*Orthogonikleithrus* n. sp. 2
	*Leptolepides* n. sp.
	*Tharsis* n. sp.

The large number of fossil actinopterygians in the lower part of the quarry (Plattenkalks I–III) containing the small teleost *Orthogonikleithrus hoelli* as prey (Tables 3–4) provides direct ‘Category 1’ evidence proving that this fish was a major food source during the early period of deposition at Ettling. Furthermore, the extreme abundance of this species at Ettling (representing 91% of fish specimens from Plattenkalks I–III) strongly suggests that its population was large at the time of deposition of the lower Ettling strata, outnumbering other fish taxa by far. These two factors, combined with its size (the smallest known fish species in the Solnhofen Archipelago, at 5.5 cm maximum length [12]), are consistent with placing *O. hoelli* near the bottom of the documented trophic hierarchy of fishes during early Ettling times. The discovery of several specimens of *O. hoelli* containing conspecific individuals suggests that this species had at least a partly piscivorous diet (occasionally engaging in cannibalism), whereas its predation on organisms at lower trophic levels remains undocumented.

Other small fishes that preyed on *Orthogonikleithrus hoelli* during early Ettling times included at least one of two other species of *Orthogonikleithrus* (*Orthogonikleithrus* n. sp. 1), and a small undescribed teleost (Teleostei n. gen. n. sp. 1). Mid-size and larger predators known from ‘Category 1’ evidence to have preyed on *Orthogonikleithrus hoelli* during the early period of Ettling deposition included *Thrissops* cf. *formosus*, *Belonostomus* cf. *kochi* and *Bavarichthys incognitus*. Interestingly, among the species known from direct evidence to have preyed on *O. hoelli* are those with the highest relative abundances at Ettling. Excluding the 3,000 known specimens of *Orthogonikleithrus hoelli* itself, these are: Teleostei n. gen. n. sp. 1 (comprising 29% of remaining fish specimens); *Orthogonikleithrus* n. sp. 1 (19%); *Thrissops* cf. *formosus* (14%); and *Belonostomus* cf. *kochi* (6%) (see Table 1). However, it is also possible that this is an artifact of preservation (because chances of finding examples of ‘frozen behavior’ increase as more specimens are found).

In addition to direct ‘Category 1’ fossil evidence, ‘Category 2A’ evidence [82] (presence of both predator and presumed prey, based on evidence from other localities) is also available for Ettling. In the Eichstätt/Solnhofen region, *Leptolepides sprattiformis* is the most common small fish species, and was placed near the bottom of the Solnhofen trophic hierarchy of fishes (with *Tharsis*) by Maisey [81]. Whereas at Eichstätt and Solnhofen *Thrissops* preyed on *Leptolepides*, in Plattenkalks I–III at Ettling *Thrissops* preyed on *Orthogonikleithrus hoelli*. It is unknown what *Leptolepides* preyed on at Ettling (as its stomach and pharyngeal contents remain unknown), although this taxon has been considered by previous authors as a plankton feeder that swam in large shoals. The lack of fine, long gill rakers and other filter-feeding adaptations suggests that fishes such as *Leptolepides*, *Orthogonikleithrus hoelli*, and *Tharsis* were not filter-feeders, but rather micro-carnivores/visual zooplanktivores and opportunistic piscivores (e.g., *Orthogonikleithrus hoelli*) that selected individual prey items in the water column and engulfed them using oral suction (M. Wilson, pers. comm.).

Of the genera illustrated by Maisey [81] in his Solnhofen trophic web, two (*Thrissops* and *Aspidorhynchus*) are present in the lower part of Ettling, and four (*Thrissops*, *Allothrissops*, *Leptolepides*, and *Tharsis*) are present in the upper part. *Thrissops* likely occupied an intermediate trophic level at Ettling, as in other Solnhofen localities (e.g., [81], [83]). According to Maisey [81], *Thrissops* and *Allothrissops* preyed on *Leptolepides* at Solnhofen localities, and this may also have been the case in the upper period at Ettling, although this is not yet proven by direct (‘Category 1’) evidence.

Although evidence of caturids from Ettling is currently limited to an isolated tooth and a fragmentary specimen, these large, sharp-toothed fishes were top predators in other localities within the Bavarian Upper Jurassic [81], [83] including Eichstätt, Solnhofen, and Painten, where they consumed the intermediate-sized fishes *Anaethalion*, *Pholidophorus*, *Ascalabos*, *Eichstaettia*, and the small teleost *Leptolepides* [81], [83]. However, due to the poverty of fossil evidence, it is unknown if caturids played as significant a role in the Ettling food web as they did at other localities in the region.

‘Category 2B’ evidence [82] (i.e., evidence inferred from tooth morphology) is also present at Ettling. Pycnodontiforms were present in both early and late Ettling times, and possessed a battery of crushing teeth appropriate for feeding on hard-shelled prey (e.g., mollusks). There is evidence that pycnodontiforms preyed on ammonites at some localities [84]. There is also direct evidence that pycnodontiforms preyed on corals, echinoids, bivalves, gastropods, decapods, and small actinopterygians ([47], [54], [85], and M. Ebert, pers. obs), although some taxa were apparently highly specialized to certain prey items. This could be inferred to provide ‘Category 2A’ evidence for similar habits among pycnodontiforms at Ettling. Echinoids, brachiopods, bivalves, gastropods, decapods, and small fishes were certainly present at Ettling, although which of these formed the primary diet of the Ettling pycnodontiforms has not yet been indicated by ‘Category 1’ evidence. Corals were presumably present in the nearby reefs, but no coral remains were found in any coprolites. However, crushed echinoid and ophiuroid remains are abundant in coprolites from Ettling Plattenkalk V. That pycnodontids were preyed on at Ettling is indicated by the discovery of at least one small coprolite (JME-ETT1895) containing the toothed prearticular of a pycnodont resembling *Turbomesodon relegans*.

Based on the above categories of available evidence, as well as comparison with closest living relatives (e.g., *Amiopsis*/living *Amia*) and the few faunal studies from other plattenkalk basins (e.g., [81], [82], [83]), Table 5 summarizes the probable trophic levels and feeding strategies of the Ettling actinopterygians. ‘High-level predators’ are here interpreted as comparatively large fishes with large, sharp, robust teeth, large gape size, and containing fossil prey fishes. Large piscivorous predators from Ettling capable of attacking larger prey include *Aspidorhynchus sanzenbacheri* and the halecomorphs *Amiopsis lepidota, Ionoscopus* sp., *Furo muensteri* (although at Ettling these taxa have not yet been found with stomach or pharyngeal contents), and possibly caturids. ‘Mid-level predators’ are intermediate in these respects, with intermediate body size, sharp but comparatively smaller teeth, and containing fossil prey fishes. ‘Low-level predators’ have a small body size, small sharp teeth, and contain fossil prey fishes of small size. ‘Durophages’ have a grinding or crushing dentition, with rounded peg-like teeth anteriorly and broader, blunt, button-shaped teeth posteriorly. Where available, stomach contents include echinoderms and other invertebrate fragments. Fishes listed as ‘Other’ in Table 5 lack teeth, and fossil stomach contents are generally absent (although occasional cannibalism has been documented in *Orthogonikleithrus hoelli* [16]). These fishes (including *O.hoelli*, *Leptolepides*, and *Tharsis*) likely represent micro-carnivores/visual zooplanktivores and opportunistic piscivores, which captured individual small prey items using oral suction (M. Wilson, pers. comm.). A diet of mainly zooplankton and small soft-bodied invertebrates would explain the usual lack of visible fossil stomach contents in these taxa.

**Changes in the Ettling fauna through time.** Geological and paleontological evidence from Ettling suggests that significant paleoecological changes occurred in the basin between the time of deposition of Plattenkalks I–III and that of Plattenkalks IV–V. Based on direct fossil evidence of predation, it can be inferred that *Orthogonikleithrus hoelli* was a major component of the fish trophic web in early Ettling times. In the lower part of the Ettling basin, *Orthogonikleithrus hoelli* appears to have populated the surface waters in great numbers, and was the main food source for a diverse array of large and small predatory fishes. The reason for the unusually high abundance of *O. hoelli* in the lower part of the Ettling quarry is unknown and remains an interesting topic for future research. By the time of deposition of Plattenkalks IV–V, *O. hoelli* had disappeared locally, and a new faunal assemblage more closely resembling that of the Eichstatt and Solnhofen basins had developed. This faunal change was accompanied by geological evidence indicating a change in depositional environment (i.e., from a calcite-based to a calcite-plus-aragonite-based primary sediment), suggesting an influx of outside sediment consistent with gradual opening of a previously isolated basin (Fellner, pers. comm., 2011; [24]).

Based on the evidence to date, Plattenkalk V also appears to contain a higher relative abundance of reef-dwelling fishes (e.g., pycnodontids, *Macrosemius*, *Macrosemimimus*) than do Plattenkalks I–III (Fig. 3, lower chart). In Plattenkalks I–III, approximately 1% of specimens (25% of species) are reef-dwellers, whereas at Plattenkalk V reef-dwellers comprise approximately 8.7% of specimens (30% of species). Numerous accumulations of disarticulated and broken echinoids and ophiuroids in the same beds (interpreted as coprolites or food remains; Fig. 4A) also suggest predation by durophagous reef-dwelling fishes (e.g., the pycnodontiforms *Turbomesodon* and *Proscinetes*; Table 5). Although these two genera also occur in Plattenkalks I–III, similar coprolites or food remains attributable to them are unknown there. The Ettling basin was surrounded by several coral reefs of unknown relative age, such as Großmehring (Late Kimmeridgian: G. Schweigert, pers. comm.), Oberdolling, Lobsing, Marching and Wackerstein [22], [32], which could be related to the occurrence of reef fishes. The occurrence of horseshoe crabs (e.g., *Mesolimulus*), and a more frequent occurrence of plant remains as well as brachiopods and bivalves, in Plattenkalk V (compared to the deeper quarry levels) is another marked difference between the upper and lower parts of the quarry, which could indicate increased proximity to land. The fossil record at Ettling, with its uniquely high proportion of actinopterygian fishes and well-preserved evidence of ancient predatory behavior, distinguishes this locality from other plattenkalk basins of the Upper Jurassic Solnhofen Archipelago and provides a rare glimpse into an ancient aquatic ecosystem of the Late Jurassic.

## Materials and Methods

All necessary permits were obtained for the described field studies by the Jura-Museum Eichstätt from the Marktgemeinde Pförring (village council of Markt Pförring, Bavaria) and its mayor Bernhard Sammiller, owners of the Ettling quarry. Specimens described herein were collected at Ettling and are reposited in the JME collections. Nearly all specimens were hand-prepared by one of us (ME). Air-pressure vibration tools and acid preparation techniques were not used, as these are unsuitable for the soft and porous Ettling stone. Moreover, acid preparation can create an artificial bias in data on fossil stomach contents, as it destroys carbonate-based material (e.g., invertebrate shells) but preserves phosphate-based items such as bone [81].
